# The “Wholesome Contact” non-pharmacological, volunteer-delivered multidisciplinary programme to prevent hospital delirium in elderly patients: study protocol for a randomised controlled trial

**DOI:** 10.1186/s13063-018-2781-6

**Published:** 2018-08-14

**Authors:** Karolina Piotrowicz, Krzysztof Rewiuk, Stanisław Górski, Weronika Kałwak, Barbara Wizner, Agnieszka Pac, Michał Nowakowski, Tomasz Grodzicki

**Affiliations:** 1 Department of Internal Medicine and Gerontology, Faculty of Medicine, Jagiellonian University Medical College, Śniadeckich 10 Str, 31-531 Kraków, Poland; 2 Department of Medical Education, Faculty of Medicine, Jagiellonian University Medical College, Łazarza 16, 31-530 Kraków, Poland; 3 Department of Health Psychology, Institute of Psychology, Jagiellonian University, Ingardena 6, 30-060 Kraków, Poland; 4 Epidemiology and Preventive Medicine, Faculty of Medicine, Jagiellonian University Medical College, Kraków, Poland

**Keywords:** Confusion, Cognitive impairment, Interdisciplinary approach, Student, Psychology students, Nursing students, Volunteering, Service learning

## Abstract

**Background:**

In hospital settings, delirium affects as many as 50% of older patients, aggravating their symptoms and worsening their condition, and therefore increasing the risk of in-hospital complications and death. The aim of this study is to assess the efficacy of structured, non-pharmacological care, delivered to older hospitalised patients by trained volunteers (students of medical fields), on the reduction of incidence of adverse health-related outcomes.

**Methods/design:**

This trial will be a randomised, investigator-blind, controlled trial conducted in an internal medicine and geriatric ward in Poland. We aim to include 416 patients who are 70 years of age and have been hospitalised for medical reasons. Eligible patients will be randomised 1:1 to receive structured, non-pharmacological care delivered by students of medicine, psychology and nursing, together with standard medical treatment or standard medical care alone. The protocol of interventions has been designed to cover nine main risk factors for delirium, with the scope of multidisciplinary interventions being individualised and tailored. The protocol will be aimed at immobilisation, vision and hearing impairment, cognitive impairment and disorientation, stress and anxiety, sleep–wake cycle disturbances, dehydration and malnutrition, and pain. A structured evaluation of patients’ cognition, mood, anxiety and functional performance is planned to be carried out twice, on the day of group allocation and at discharge; structured screening assessment for delirium will be conducted daily using the Confusion Assessment Method. The primary outcome will be the incidence of delirium in hospital; secondary outcomes will be in-hospital changes in cognition, mood and anxiety, and functional status, occurrence of falls and death.

**Discussion:**

Delirium prevention programmes are being introduced worldwide. A particular novelty of our project, however, is that invitations for voluntary work with older patients at risk for delirium will be addressed to medical students. With the use of the service learning method, the students will shape their attitudes, increase their knowledge and understanding of hospital care, and master competencies to work within interdisciplinary teams, which establishes the originality and practicality of the project.

**Trial registration:**

Polish Science Database, 317484. Registered on 23 October 2016.

**Electronic supplementary material:**

The online version of this article (10.1186/s13063-018-2781-6) contains supplementary material, which is available to authorized users.

## Background

Delirium is an acute, fluctuating and potentially reversible change in a person’s awareness, cognition and behaviour [1, 2]. Its incidence has been linked to a range of different risk factors [1, 3]. The prevalence of delirium has been shown to increase with advancing age, and is related to burdening comorbidity and multimorbidity, an accumulation of deficits or disabilities, and unfavourable environmental conditions and psychological stress [1, 3].

In hospital settings, delirium affects as many as 50% of vulnerable older adults, prolonging their hospitalisation period, aggravating their symptoms and worsening their condition, and therefore increasing the risk of in-hospital death or the need for further post-hospital care with total dependence on basic and instrumental activities of daily living along with the inevitability of institutionalisation [1, 4, 5]. Given that delirium is one of the most common and harmful geriatric threats, its early recognition and effective management is still largely neglected or missed by staff on duty [5].

The idea of non-pharmacological prophylaxis and treatment of hospital delirium was pushed for and then put into action by Prof. Sharon Inouye in the late 1990s, with the Hospital Elder Life Program (HELP) for Prevention of Delirium [6]. The initial project was comprised of six highly tailored interventions targeting cognitive impairment, sleep deprivation, immobility, visual and hearing impairment, and dehydration [7]. Many programmes inspired by HELP have since been implemented [8–12]. A systematic overview of systematic reviews on the effectiveness of the non-pharmacological approach to delirium in older patients, published in the framework of the SENATOR Project ONTOP Series, has proved the efficacy of multicomponent non-pharmacological preventive strategies for reduction of delirium incidence, for interventions conducted in medical and surgical wards [13].

Despite the fact that the high effectiveness and cost-efficiency of such comprehensive, multicomponent, patient-centred strategies has been proven, a worldwide protocol is yet to be established [5, 14, 15].

### Rationale for proposed study

Based on a review of the literature, the barriers and difficulties that might limit the effective implementation of a non-pharmacological multicomponent method of preventing hospital delirium have been recognised [5–15]. Besides the compelling need for formalised protocols, the shortage of geriatricians and the forecast shortfall of nurses and medical assistants, general overworking and burnout among medical staff that might lead to a reluctance to take on new responsibilities, obligations and duties, as well as financial limitations, were identified as potential problems.

As a unique solution in the face of these difficulties, a group of medical and psychology student volunteers was invited to participate on a partnership basis in our programme of non-pharmacological prevention of in-hospital complications.

We searched Medline and Google databases using the keywords and MeSH headings for delirium, non-pharmacological prevention, students and volunteers, as well as Cochrane reports, and the retrieved articles were browsed manually, but no results were obtained except for a single original report on a pilot study of our project [16]. Therefore, we can say that our programme is particularly novel in that it invites students to participate in delirium prevention. It is the first time that medical, psychology and nursing students specifically have been invited to participate in voluntary work with older patients at high risk for delirium.

What is more, and of particular importance for academic institutions, when designing and then launching the “Wholesome Contact Project” we availed ourselves of the opportunity to provide the students engaged in the project with the novelty of a service learning method. The service learning method is experimental training, based on the principle of equal benefits for both the recipients and the providers of the service [17]. In this manner, the students will deliver non-pharmacological care for older patients and at the same time shape their own attitudes towards older and disabled people, increase their knowledge and understanding of hospital care, and master their competencies to work in interdisciplinary teams.

### Objectives

The aim of our study is to assess the effect of structured non-pharmacological care delivered by trained volunteers (medical, psychology and nursing students) on older patients hospitalised in the internal medicine and geriatric ward, in relation to incidences of adverse health-related outcomes such as delirium, falls and in-hospital deaths, in-hospital changes in cognition, mood and anxiety, and functional status, in comparison to standard medical care alone.

We hypothesised that a structured, multidisciplinary, volunteer-delivered non-pharmacological programme of in-hospital delirium prevention will reduce the rate of adverse health outcomes, with particular influence on the incidence of delirium.

## Methods

### Study design

**Fig. 1:**
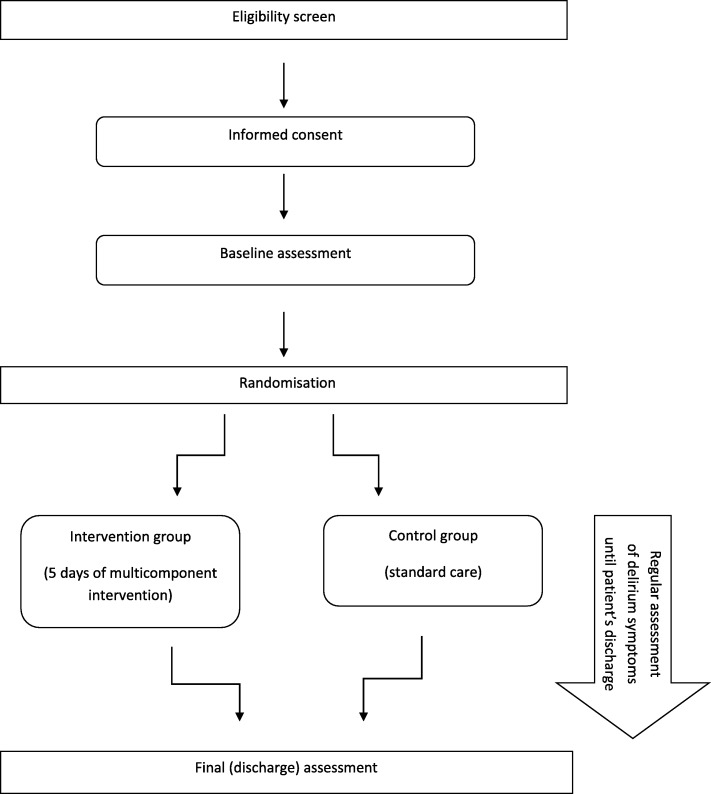
Flow diagram of the study

A randomised, blind, controlled trial, allocated at a ratio of 1:1, is being planned to start, in order to show the superiority of multidisciplinary, structured care delivered by medical, psychology and nursing students together with standard medical treatment over standard medical care alone (Fig. 1).

### Eligibility criteria

Our inclusion criteria will be: age ≥ 70 years, acute admission to hospital, ability to communicate and maintain logical verbal contact at the time of group allocation, and patient’s informed consent.

Our exclusion criteria will be: delirium or psychotic disorder at the time of admission, severe cognitive impairment (defined as Mini-Mental State Examination (MMSE) ≤ 9 points) [18, 19], terminal condition and transfer from another ward or hospital.

### Study setting

This is a single-centre study that is to be conducted in the Department of Internal Medicine and Geriatrics at the teaching hospital of Jagiellonian University in Kraków.

Some unfavourable environmental factors that may contribute to in-hospital adverse health-related outcomes are perceptible in our setting (e.g. customisation of beds that are placed in the corridor, used temporarily in the event of a sudden influx of new patients, as well as crowded, four and five-bed rooms).

All rooms are equipped with wall calendars and clocks. The lighting system is universal, but adaptable to maintain low light at night (from 22:00 to 06:00), except in life-threatening situations. Maximum recommended night-time illumination should be no stronger than 100 lx, checked with a handheld light meter (Standard ST-1301). No protocols have yet been introduced to reduce hospital noise or to correct a lighting system unadjusted to day and night time.

A high number of medical staff (on average, eight doctors, six nurses and two nursing assistants on day shift, and two, three, and one of them on night shift, respectively) and their substantial turnover, without nurses being assigned to a particular patient for the period of hospitalisation, is noticeable.

Visiting hours are from 14:00 to 20:00, but are flexible in some instances. Generally, caregivers and proxies are encouraged and invited to spend as much time with their relatives as possible.

### Interventions

#### Intervention group

Patients are to be visited by volunteers for the first 5 working days of their hospitalisation. An initial visit is being planned to be undertaken within the first 24 h of hospitalisation if a patient has been admitted from Sunday at noon to Friday at noon. Otherwise, the timeframe for the first meeting with volunteers is 72 h.

The first visit (called the familiarisation process) will be made by two specific volunteers assigned to the patient, and the subsequent visits by one of them. Patients will be assigned to these particular visitors based on a list of available volunteers.

Our assumption that patients should be tracked for the first 5 days of their hospital stay is derived from the findings that delirium occurs most frequently during the first days of hospitalisation, and from the practical approach in which parts of the intervention planned are deemed profitable only at the beginning of a hospital stay (e.g. acquainting with the spatial organisation of hospital rooms, presenting available services, calling for hearing aids, glasses, dentures, walking aids, clocks and calendars) [20].

It must be emphasised that volunteers will be engaged not to substitute for all of the regular medical care workers, but to amplify the positive effect of non-pharmacological and pharmacological treatment that all patients will receive according to the “Evidence-Based Medicine” and “Good Clinical Practice” guidelines. Neither the administration of drugs nor pharmacological or non-pharmacological interventions will be prohibited during the study.

The protocol of interventions has been designed to cover nine main risk factors for delirium incidence (“The Volunteer–Patient Contact Form” has been published elsewhere [16] and is included in Additional file 1: Table S1). “The Volunteer–Patient Contact Form” is an observation card (chart) designed to allow a pair of volunteers to follow a standardised method of care procedure and to adhere to the protocol, with all nine risk factors being covered daily (see Additional file 1: Table S1).

All interventions within the trial protocol are non-invasive and harmless. Nevertheless, information on the adverse events that potentially might arise from implementation of the protocol is included, and will be repeated during the training sessions (i.e. falls, injuries, fluid and food aspiration). In such an event, the volunteers will be required to inform medical staff immediately, and put this information in “The Volunteer–Patient Contact Form”.

Should patients or their proxies so demand, the intervention might be discontinued or optionally limited immediately, at any time, without the need for additional explanations or consequences related to further hospitalisation and treatment. All of the changes made will be recorded on patient observation cards.

For patients who drop out of the study, data on primary and secondary endpoints and time of hospital stay will be collected.

### Description of the targeted risk factors for hospital delirium

The scope of multidisciplinary intervention was established following a scrutinous review of the existing protocols for non-pharmacological approaches to delirium prevention [6–13]. It combines six risk factors included in the best-known and widely recognised HELP protocol (such as immobilisation, sensory deprivation (vision and hearing impairment), cognitive impairment and disorientation, sleep–wake cycle disturbances and dehydration) [6, 7], but as a novelty also includes malnutrition, stress and anxiety, and pain management. The overall scope of intervention will be individual and tailored at the discretion of the project coordinator (SG), and depend on the extent of the problem being addressed, adjusted daily according to each patient’s health status and comfort. There is no time limit for the visit (the volunteer should use their discretion and proceed according to “The Volunteer–Patient Contact Form”).

#### Immobilisation

Volunteers will reinforce the effort of physiotherapists working with patients regularly, and therefore encourage and support them in physical activity being undertaken between physiotherapy sessions.

Volunteers should remind patients of the health benefits that accrue from physical activity, and dispel doubts in the event of a patient’s reluctance. If necessary, they will consult physiotherapists or contact medical doctors on duty. The spectrum of activities recommended ranges from bed or chair transfer to accompanying patients when walking independently or with some walking aids.

#### Sensory deprivation—hearing impairment

Volunteers will make sure that patients’ hearing aids are accessible and work properly, if worn. It is recommended that patients’ relatives are contacted if patients’ hearing aids have been left at home. For hearing impairments without adequate correction, a specific manner of speaking will be adopted.

#### Sensory deprivation—vision impairment

Volunteers will make sure that patients’ glasses, if worn, are accessible. It is recommended that patients’ relatives are contacted if patients’ glasses have been left at home. For vision impairments without sufficient correction, a magnifying glass for reading will be offered.

#### Cognitive impairment and disorientation

All patients will be involved in activities that include some elements of cognitive stimulation training. According to preferences expressed by patients, volunteers will read them the latest newspapers and magazines, discuss recent events with them and engage them in stimulating conversation about their hobbies or leisure activities. It is intended, with this activity, to reorient patients to time, place and person if needed.

#### Stress and anxiety

Assuming a high level of emotional stress related to hospitalisation, volunteers will explain the main practical principles of the ward organisation to patients (e.g. the daily schedule), acquaint them with the spatial arrangements of the hospital environment and show them how to operate necessary medical devices. Patients will be openly questioned about their worries and fears, and encouraged to discuss their worries with their primary medical team.

#### Sleep–wake cycle disturbances

Our aim is to promote the idea of diurnal rhythms of patients’ activity in compliance with external circadian rhythms. Patients will be involved in tiring but individually tailored activities in the afternoon and early evening, and instructed to go to bed at a set time each night. If necessary, the basic rules of sleep hygiene will be presented to patients. Any complaints about sleep problems will be reported to the doctors on duty.

#### Dehydration

Volunteers will educate patients about the importance of and need for regular fluid intake. They will ensure that patients have some fluids to hand and equip them with drinking straws if needed. It is also planned to raise volunteers’ awareness, during training sessions, about swallowing problems, which should be reported to dieticians or doctors on duty.

#### Malnutrition

Volunteers might assist patients during mealtimes. Their attention will be directed to patients’ level of independence while eating (when slicing and cutting, when using cutlery), as well as to any problems with chewing, swallowing or choking. Volunteers will make sure that patients’ dentures are accessible and worn. It is recommended that patients’ proxies are contacted if patients’ dentures have been left at home. Last but not least is the aspect of enjoying the company and a pleasant and friendly atmosphere during these meals.

#### Pain

All patients will be asked about pain, and if applicable, the presence of uncontrolled pain will be reported to the nurses and doctors on duty.

### Control group

Standard, widely accepted treatment that is normally administered to patients with certain diseases, conditions or symptoms will be used in a control group. All of the patients will receive pharmacological and non-pharmacological therapy in accordance with the principles of the “Evidence-Based Medicine” and the “Good Clinical Practice” guidelines. Non-pharmacological treatment of older patients, as a rule, includes personalised elements of multidisciplinary care based on individual results of comprehensive geriatric assessment. Each and every hospitalised patient receives care from physiotherapists, nutritionists, psychologists and social workers, and from clerics if considered beneficial and preferred by them.

### Eligibility criteria and training for volunteers

The invitation has been issued to students in all medical fields, regardless of whether they are in medical or nursing school, and to students of psychology at Jagiellonian University in Kraków. All volunteer candidates must have completed at least the first year of their studies.

Before participating, all candidates are obliged to submit a letter of motivation and complete a series of training sessions.

The training sessions consist of an introductory session about the general rules and regulations of volunteering (including ethical issues, patients’ rights and medical confidentiality, with a duration of about 90 min), a session on occupational health and safety (with a duration of about 45 min) and a group training session on communication skills (about 90 min), as well as practical training on how to handle and mobilise patients safely (about 45 min), seminars and group discussions about major geriatric issues (with particular focus on symptoms of delirium—its recognition, primary and secondary prevention, falls, malnutrition and dehydration) as well as information on the adverse events that potentially might arise from implementation of the protocol (three sessions of about 60 min each). Supervision sessions, each lasting about 90 min and with an experienced psychologist (WK) as a supervisor, will be provided for all volunteers every month. Besides that, the coordinator of the project (SG) and the psychologist (WK) will be on call every day. In order to increase volunteer retention in the project, some initiatives and leisure activities are being planned in order to build and reinforce a sense of community among these volunteers.

### Outcomes

#### Primary outcome

The primary efficacy outcome will concern incidences of hospital delirium. Delirium will be diagnosed by the Confusion Assessment Method (CAM) [21]. A structured screening assessment for delirium incidence will be conducted daily on working days between 07:00 and 12:00 by one of the two researchers experienced in geriatrics available on site, unaware of the group assignment and with the use of a screening algorithm of the CAM tool—the shortened version of the CAM worksheet [21, 22]. If a diagnosis of delirium is suggested by the CAM shortened version (i.e. if an acute onset and fluctuating course, inattention, disorganised thinking and/or altered level of consciousness will be present), a complete assessment with the full CAM questionnaire will be carried out. We plan to use a Polish version of the CAM that has been translated by one of the researchers from our study team (KP) and has already been used in the project on hospital delirium, with its results published elsewhere [23, 24]. Additionally, doctors’ and nurses’ reports will be analysed. Both investigators will be blind in respect of patients’ allocation to the study groups.

The CAM questionnaire is the most widely used scale for delirium screening, assessment and diagnosis worldwide. It was invented and introduced by Prof. Sharon Inouye [21]. This instrument has been shown to demonstrate not only high sensitivity and specificity, but also high positive and negative predictive values. It is easy to perform and not time consuming, and is capable of being implemented by a non-psychiatrist. Both of the researchers have been trained to use the CAM instrument according to the Training Manual and Coding Guide [21]. Patients’ attention (focusing, maintenance and shifting of attention) will be assessed with the use of a subtask of the Mini-Mental State Examination, that is calculation (serial sevens subtraction) or spelling the word “WORLD” backwards [19, 21].

Additionally, medical staff on duty will be asked twice a day (at 7:00 and at 14:00) and medical records will be analysed for any clinical signs of delirium, followed if applicable by the structured delirium assessment (if delirium is suspected, patients will be evaluated with the shortened and the full versions of CAM, as already described). By looking through medical records and interviewing attending physicians and doctors on duty daily, detailed information on patients’ cognition will be gathered.

A 45-min training session for medical staff working on the ward, on delirium symptoms and preventive strategies, was conducted twice, at a time interval of 12 months, by one of the researchers (KP).

#### Secondary outcomes

A secondary outcome will be the following:

1. Occurrence of in-hospital adverse health outcomes, such as falls and in-hospital deaths (the number of episodes of falls will be noted in medical records, as will the number of in-hospital deaths, regardless of cause).
2. In-hospital changes (i.e. the difference noted between the day of baseline assessment and the day of discharge) in cognition (difference in the Mini-Mental State Examination (MMSE) score), mood and anxiety (difference in the Hospital Anxiety and Depression Scale score (HADS)) and functional status (difference in the Activities of Daily Living Scale (ADL) and the Instrumental Activities of Daily Living Scale score (IADL)), assessed as described in the following.

### Participant timeline and assessment tools

A structured evaluation of patients’ cognition, mood and anxiety and functional performance is planned to be done twice during the course of the study. The first baseline examination is planned to be carried out on the day of group allocation, and reassessment will be done at the time of discharge. In order to reduce the effect of diurnal variability in patients’ cognition and attention, each examination will take place between 12:00 and 14:00.

The following tools will be used in the study: the Mini-Mental State Examination for cognitive impairment [19], the Hospital Anxiety and Depression Scale for mood and anxiety assessment [25, 26], and the Activities of Daily Living Scale (Katz scale) and the Instrumental Activities of Daily Living Scale (Lawton scale) for functional status [27, 28].

The Mini-Mental State Examination was invented and introduced by Folstein et al. in 1975 [19], and is now one of the most widely used instruments for cognitive screening assessment in old age. It consists of 19 parts that examine different cognitive domains, i.e. orientation, registration and recall, attention and calculation, language (naming, repetition, comprehension and writing) and constructional praxis. It is obligatory for the interviewed subjects to be literate and fluent in the language of examination. The subjects are scored between 0 and 30 points, with the lowest results being suggestive of dementia [19].

The Hospital Anxiety and Depression Scale was introduced in 1983 by Zigmond and Snaith [25], for the evaluation of psychological distress in hospitalised patients. It comprises of two subsets of questions regarding anxiety and depression experienced by patients. The subject is assessed with the use of 14 items, each scored between 0 and 3 points, with the highest results indicating more distress [25, 26].

Functional performance will be evaluated using the Activities of Daily Living and the Instrumental Activities of Daily Living Scale [27, 28]. The ADL scale consists of items such as independence in bathing, dressing, self-feeding, functional mobility and personal and toilet hygiene [27]. The IADL scale assesses independence in using a telephone, transportation within the community, shopping for groceries, preparing meals, doing housework, laundry, and home-improvement, taking medication as prescribed and managing money [28]. Functional impairment will be identified if the patient scores ≤ 24 out of a maximum of 27 points on the IADL scale. Functional disability will be suspected if the subject scores ≤ 4 out of a maximum of 6 points in the ADL scale.

**Fig. 2:**
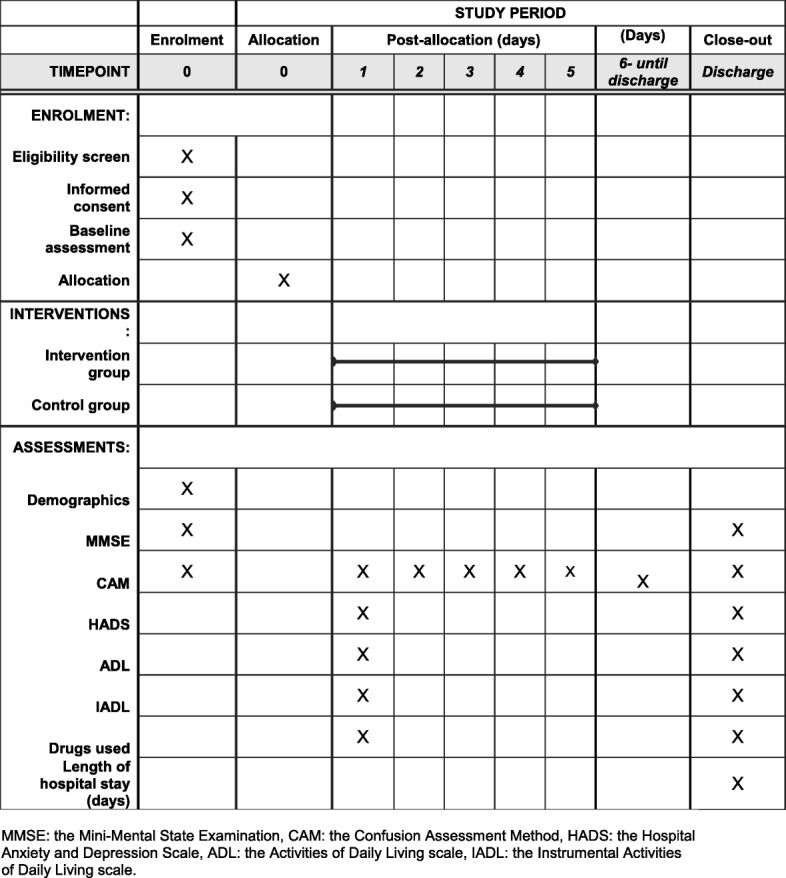
SPIRIT diagram for time schedule of enrolment, allocation, intervention and patient assessments. MMSE Mini-Mental State Examination, CAM Confusion Assessment Method, HADS Hospital Anxiety and Depression Scale, ADL Activities of Daily Living scale, IADL Instrumental Activities of Daily Living scale

A schematic diagram of the time schedule of enrolment, allocation and assessment is presented in Fig. 2, created according to the Standard Protocol Items: Recommendations for Interventional Trials (SPIRIT) requirements.

The assessors will be doctors undergoing residential training in geriatric medicine. Standardisation training will be accomplished before the study and then every 6 months during the course of the study.

In all patient demographics, information on the duration of hospitalisation and medical data will be gathered, and the Charlson Comorbidity Index will be calculated [29]. The duration of delirium (median, Q1; Q3 days) and the need for antipsychotic drugs and physical restraint (proportion of patients in whom these are administered) will also be noted.

### Participant recruitment

An invitation to the project will be addressed to all patients aged 70 years and older being admitted to the Department of Internal Medicine and Geriatrics, University Hospital in Kraków. The subjects for the project will be recruited for 2 years according to the inclusion and exclusion criteria, by one member of the study team (KP, SG). All of the recruited subjects will give their written, informed consent before taking part in the study. The rules of the study (including information that not all of the patients will be visited by volunteers) are to be explained to the patients before they give informed consent.

### Statistical considerations

#### Sample size

The sample size estimation for the study was based on the results from pilot research on delirium conducted by one of the study team (KP), published in the Polish geriatric journal [23]. According to the data obtained, it has been assumed that delirium might be experienced in about 20% of hospitalised patients who meet the inclusion and exclusion criteria for the study. Based on the literature [12], with multicomponent prophylaxis intervention we would expect an odds ratio (OR) for delirium incidence of at least 0.43. Assuming a type I error of 5% and power of 80%, 187 patients would be randomised to each group. Assuming approximately 10% drop out, 208 patients would be included in each arm of the study.

#### Allocation and blinding

The included participants will be randomly assigned to one of the two groups (intervention or control). A staff member (KR), not involved in data collection, will allocate the participants to the study or the control group by a dice-throwing technique (with stratification according to age (70–79 years and ≥ 80 years), baseline cognitive status (MMSE of 10–23 and MMSE of 24–30 points) and gender). The ratio of assignment to the each arm will be 1:1 and it is planned for each arm to be comprised of 208 individuals.

The volunteer coordinator (SG) will be informed immediately about inclusion of a new patient in any research arm. If included in the intervention arm, a minimal set of the patient data will be delivered in person, in a sealed envelope, by the coordinator to the first available pair of volunteers. The volunteers will be listed and referred to thereafter with regard to their initials. In this manner, investigators will be blind to the patients’ allocation until hospitalisation ends or a primary endpoint (delirium) occurs. In the event of a diagnosis of delirium, the study coordinator will be informed and an unblinding procedure will take place. For safety reasons (e.g. hyperactive delirious patients that might threaten substantial harm to themselves or others), further delirium treatment will be led by attending medical staff.

Practical information on how to ensure the blinding of investigators is included in the materials and slides that have been prepared for the standardisation training of the assessors. The assessors will be instructed not to discuss the study issue with patients (if needed, the coordinator will be called). If patients introduce this subject spontaneously, the investigators will politely attempt to change the topic and be aware that the patients to be included in the study might be visited not only by student-volunteers, but also by regular students during their rotation at the University Hospital.

#### Statistical analysis

The incidence of primary outcome, that is delirium, as well as the secondary outcomes, such as falls or all-cause mortality, will be compared between the intervention and standard care groups using chi-square statistics. To assess the statistical difference between the intervention and control groups in terms of mood and anxiety levels, cognitive and functional status (e.g. HADS, MMSE, ADL and IADL scores) or length of stay, the Student’s *t* test or Mann–Whitney *U* test will be used, and the normality of distribution will be tested using the Shapiro–Wilk test. The parameters of descriptive statistics (arithmetic mean or median, standard deviation or interquartile range, and percentage distribution of qualitative variables) will present baseline characteristics of the compared groups. Regression analyses (logistic or standard) will be performed to assess the size effect of the intervention in prevention of delirium incidence, as well as other health-related adverse incidences (falls, death, decline in cognitive function, mood and anxiety, and functional status). Kaplan–Meier survival analysis will be carried out to describe the time function to assess the probability of delirium, fall and all-cause mortality in the intervention group and the standard care group.

### Ethics, consent and permissions

The study has been endorsed by the Ethics Committee of Jagiellonian University, Kraków, Poland (number 122.6120.18.2015). All of the recruited subjects will give their written, informed consent before taking part in the study. If applicable, any important modifications to the study protocol and adverse events will be reported to the Ethics Committee of Jagiellonian University in annual reports.

All of the items addressed in the presented trial protocol were covered and checked according to the SPIRIT checklist (Additional file 2: Table S2).

## Discussion

The idea of volunteer-based care for elderly inpatients was launched in 1993 by Prof. Inouye at Yale University School of Medicine [6]. The efficacy of the prototype concept of non-pharmacological, multidisciplinary prevention of delirium delivered by trained volunteers, comprising six targeted risk factors such as cognitive impairment, sleep deprivation, immobility, dehydration and visual and hearing impairment, has proven not only to reduce the number and duration of delirium episodes, but also cost-effectiveness, sustainability and scalability [7, 10, 11]. Henceforth, delirium prevention programmes and initiatives aimed at better quality of care, encouraged by these results, have been introduced and set up [4–7]. However, all of the programmes available engage trained volunteers who are managed (educated and supervised) by a contracted volunteer coordinator [8–12, 30].

It is widely known that adequate knowledge in the field of delirium is lacking among both non-geriatric and geriatric specialists [31–34]. As shown in the research by Bellelli et al. [32], only 84.5% of doctors, 49.8% of nurses, 52.9% of physiotherapists and 76.7% of psychologists affiliated to the Italian Association of Psychogeriatrics (IAP) correctly defined delirium. Lack of awareness of the problem of delirium prevalence, diagnosis and appropriate treatment was also revealed in the survey by Davis and MacLullich [33], conducted in 2009 among 784 respondents from 34 hospitals across the UK. Furthermore, only 30% of interviewed participants confirmed their confidence when managing delirium. While rising awareness of this problem via delirium advocacy campaigns has been noted [34], practical skills and attitudes remain neglected [35]. Fisher et al. [35] showed in a study carried out from October 2013 to March 2014 at 24 UK medical schools that 19 of them reported teaching outcomes related to the skills, and only two of them related to attitudes towards patients suffering from delirium. Unfortunately, no study on delirium problem awareness has so far been conducted among Polish practitioners and medical schools. While our project in its current form, with the presented study protocol, is not disposed to evaluate the students’ awareness of delirium, if successful it nevertheless seems rational to extend it by evaluating students’ knowledge, awareness and competencies in geriatric medicine in the future.

A particular novelty introduced in our programme is that the invitation to participate in the project is addressed to medical, psychology and nursing students. With a concise, easy-to-perform, replicate and follow nine-item protocol of non-pharmacological prevention of in-hospital complications, designed to by carried out by student volunteers, we hope to demonstrate the efficacy of a costless, preventive approach to delirium, which is one of the most serious geriatric problems. If this project is successful, we hope to launch an initiative that will foster not only better quality of care for older hospitalised adults, but also lead to further cooperation between geriatric units and academic settings. ICT technologies make it possible to employ simulation-based training or e-learning modules to increase students’ competency in geriatrics and gerontology satisfyingly [36, 37]. Nonetheless, the acquisition of soft skills requires a longer training period, higher frequency and deeper personal and emotional involvement, often with tedious efforts [38, 39]. By inviting student volunteers to participate in shared care delivered for older hospitalised patients, in exchange for creating for them new learning opportunities, we would be able to obtain additional, costless, voluntary service provided by medical students. In the face of the inevitable shortfall in qualified and experienced medical staff, it seems of great importance to design for them new educational alternatives with modern and efficient techniques, allowing them to learn how to communicate effectively, work in interdisciplinary teams, obtain long-lasting knowledge and understanding, and, last but not least, improve their capacity to work competently in different clinical settings. Therefore, with the implementation of the principles and rules of the service learning that we hope to employ, we recognise not only the originality and novelty of our project, but also its practical feasibility. In these circumstances, expected and anticipated limitations might arise, presumably from a lack of dialogue, understanding and cooperation between the stakeholders who should be involved; that is, academic and hospital settings and their authorities.

The results of our pilot study have been published elsewhere [16, 40, 41]. In the retrospective analysis of the medical records of 130 patients included in the pilot study, we showed that implementation of our non-pharmacological interventions, delivered by the trained volunteers, reduced the need for antipsychotic medications and shortened the length of hospital stay [16]. Last but not least, this was a longitudinal qualitative study with an idiographic approach on the experience of the eight volunteers, engaged in the pilot phase of the project, which aimed to describe their changes in experience, beliefs, motives and self-understanding [41].

We are aware of some limitations to our study protocol. First, when analysing data it seems impossible to gather and take into account all potential confounding factors. As we plan to work with the most heterogeneous group of patients, each and every one of them will present a unique cluster of comorbidities, symptoms and geriatric problems that are immeasurable and incomparable. However, we intend to conduct some elements of comprehensive geriatric assessment and to quantify the multimorbidity burden with some indices (including the Charlson Comorbidity Index) [29]. Second, despite best efforts, we are not able to control the hospital environmental conditions and related psychological stress (e. g. sensory deprivation, sleep–wake cycle disturbances due to the use of strong-light during sleeping hours, relocation of patients within the ward) that might have negative health outcomes, including delirium, and therefore potentially induce some bias. What is important, however, is that the hospital environment is expected to be the same for the intervention and the control groups, with significant differences stemming from the setting and patients’ surroundings not foreseen. Last but not least, due to the wide implementation of the project so far, the medical staff have already been sensitised not only to delirium symptoms, but also to the preventive strategies that might result in non-pharmacological prophylaxis being randomly introduced to the patients from the control group.

However, it seems of particular importance that the project setting is highly representative for a wide range of noxious delirium risk factors, presented and recognised in many clinical settings worldwide.

### Trial status

Patient recruitment is to be started from May 2018; the planned time frame for data collection is 24 months of volunteers’ contributions.

## Additional files

Additional file 1: **Table S1.** Volunteer–Patient Contact Form (DOCX 18 kb)

Additional file 2: **Table S2.** SPIRIT checklist (DOCX 61 kb)

## Abbreviations

ADL: Activities of Daily Living scale
CAM: Confusion Assessment Method
HADS: Hospital Anxiety and Depression Scale
IADL: Instrumental Activities of Daily Living scale
MMSE: Mini-Mental State Examination
