# Supplementary material for: The “Wholesome Contact” non-pharmacological, volunteer-delivered multidisciplinary programme to prevent hospital delirium in elderly patients: study protocol for a randomised controlled trial
Source: Trials. 2018 Aug 14;19:439. doi: 10.1186/s13063-018-2781-6 (PMC6092850; doi:10.1186/s13063-018-2781-6)
Supplement: Supplementary file 1 — Table S1. Volunteer–Patient Contact Form (DOCX 18 kb) [file 13063_2018_2781_MOESM1_ESM.docx]

**Additional file 1**

Table S1. The Volunteer-Patient Contact Form

The Volunteer-Patient Contact Form

| Volunteer’s name | | | | | | | | | | | | | | | |
| --- | --- | --- | --- | --- | --- | --- | --- | --- | --- | --- | --- | --- | --- | --- | --- |
|  | | |  | |  |  | |  | |  |  | |  |  |  |
|  | | |  | |  |  | |  | |  |  | |  |  |  |
| Date  Risk factors |  |  | |  | | |  | |  | | | Intervention examples | | |  |
| Immobilisation |  |  | |  | | |  | |  | | | A short walk with the patient, simple exercises in bed. | | |  |
| Sensory deprivation – hearing impairment |  |  | |  | | |  | |  | | | Speaking loudly and making sure that the patient can hear well.  If the patient is using a hearing aid, checking whether it is turned on and has working batteries.  Asking patient’s family to bring the hearing aid if it was left at home. | | |  |
| Sensory deprivation – vision impairment |  |  | |  | | |  | |  | | | Checking if the patient needs glasses and has them with them.  Asking patient’s family to bring the glasses  if they were left at home.  Offering a magnifying glass for reading. | | |  |
| Cognitive impairment  and disorientation |  |  | |  | | |  | |  | | | Chatting about the patient’s interests and current affairs.  Bringing a newspaper. Providing information about current time and place. | | |  |
| Stress and anxiety |  |  | |  | | |  | |  | | | Encouraging the patient to share their concerns with hospital personnel.  Explaining to the patient how to operate a bed remote control and a buzzer summoning a nurse, providing information on daily routine on the ward, meals and doctors’ rounds schedule. | | |  |
| Sleep-wake cycle disturbances |  |  | |  | | |  | |  | | | Taking care of the patient’s circadian rhythm – encouraging the patient to avoid sleeping during the day, so that they can sleep better at night. If noise is an issue, advising family on bringing earplugs for the night. | | |  |
| Dehydration |  |  | |  | | |  | |  | | | Making sure that the patient has enough liquids at hand, supplying some if there are not any.  Paying attention to whether the patient is able to drink independently. If needed, assisting with drinking, e.g., by passing a straw. | | |  |
| Malnutrition |  |  | |  | | |  | |  | | | Checking whether the patient uses dentures and has them – if not, intervening to provide them.  Assisting the patient with eating, e.g., visit during meal times.  Passing any information about patient’s difficulties with food intake to the hospital personnel. | | |  |
| Pain |  |  | |  | | |  | |  | | | Asking about pain, and if present, reporting it to the nurses and doctors on duty. | | |  |

**Please remember to put down information if any adverse event occurs during the intervention (be specific about what happened and when).**
